# Evaluation of Dynamic Tumor-tracking Intensity-modulated Radiotherapy for Locally Advanced Pancreatic Cancer

**DOI:** 10.1038/s41598-018-35402-7

**Published:** 2018-11-20

**Authors:** Akira Nakamura, Masahiro Hiraoka, Satoshi Itasaka, Mitsuhiro Nakamura, Mami Akimoto, Yoshitomo Ishihara, Nobutaka Mukumoto, Yoko Goto, Takahiro Kishi, Michio Yoshimura, Yukinori Matsuo, Shinsuke Yano, Takashi Mizowaki

**Affiliations:** 1 Department of Radiation Oncology and Image-Applied Therapy, Graduate School of Medicine, Kyoto University, Kyoto, Japan; 2 Department of Radiation Oncology, Kurashiki Central Hospital, Kurashiki, Japan

## Abstract

Intensity-modulated radiotherapy (IMRT) is now regarded as an important treatment option for patients with locally advanced pancreatic cancer (LAPC). To reduce the underlying tumor motions and dosimetric errors during IMRT as well as the burden of respiratory management for patients, we started to apply a new treatment platform of the dynamic tumor dynamic tumor-tracking intensity-modulated radiotherapy (DTT-IMRT) using the gimbaled linac, which can swing IMRT toward the real-time tumor position under patients’ voluntary breathing. Between June 2013 and March 2015, ten patients were treated, and the tumor-tracking accuracy and the practical benefits were evaluated. The mean PTV size in DTT-IMRT was 18% smaller than a conventional ITV-based PTV. The root-mean-squared errors between the predicted and the detected tumor positions were 1.3, 1.2, and 1.5 mm in left-right, anterior-posterior, and cranio-caudal directions, respectively. The mean in-room time was 24.5 min. This high-accuracy of tumor-tracking with reasonable treatment time are promising and beneficial to patients with LAPC.

## Introduction

Intensity-modulated radiotherapy (IMRT) can provide an excellent dose distribution for the most complex cancer volumes and has the potential to drastically increase the therapeutic ratio when the target tumor is immobile. However, the advantages of IMRT may be impaired unless the organ motion is addressed, because underdosing the target or overdosing the normal tissue can occur from organ motion during beam delivery and throughout the treatment course^1^. Recently, four-dimensional radiotherapy (4DRT) has been realized with technical innovations by adapting the treatment beam directly to the real-time moving target of thoracic and abdominal tumors^2–7^. Incorporating 4DRT into IMRT can reduce the uncertainties of IMRT for moving tumors. Various intractable cancers, such as pancreatic ductal adenocarcinoma, can be the target of this strategy.

Pancreatic ductal adenocarcinoma is among the most devastating malignant diseases, which is illustrated by its high mortality rate and its equally high incidence^8^. Although controversy exists in the role of conventional RT for unresectable locally advanced pancreatic cancer (LAPC)^9^, IMRT is now regarded as an important option for reducing doses to the adjacent organ-at-risk (OAR) and improving local control in LAPC^10–13^. Because respiration is a major cause of pancreas movement^14,15^, the breath holding or respiratory gating technique during IMRT is recommended and widespread^16^. However, growing evidence suggests that these modern techniques are still suboptimal because of the residual motion during the breath-hold^17,18^ or the volatility in internal/external correlation used for respiratory gating^19^. These uncertainties can yield a discrepancy between planned and delivered doses, and therefore reduce the advantage or safety of IMRT. In this context, introducing a 4DRT technique has the potential to refine pancreatic IMRT.

**Figure 1:**
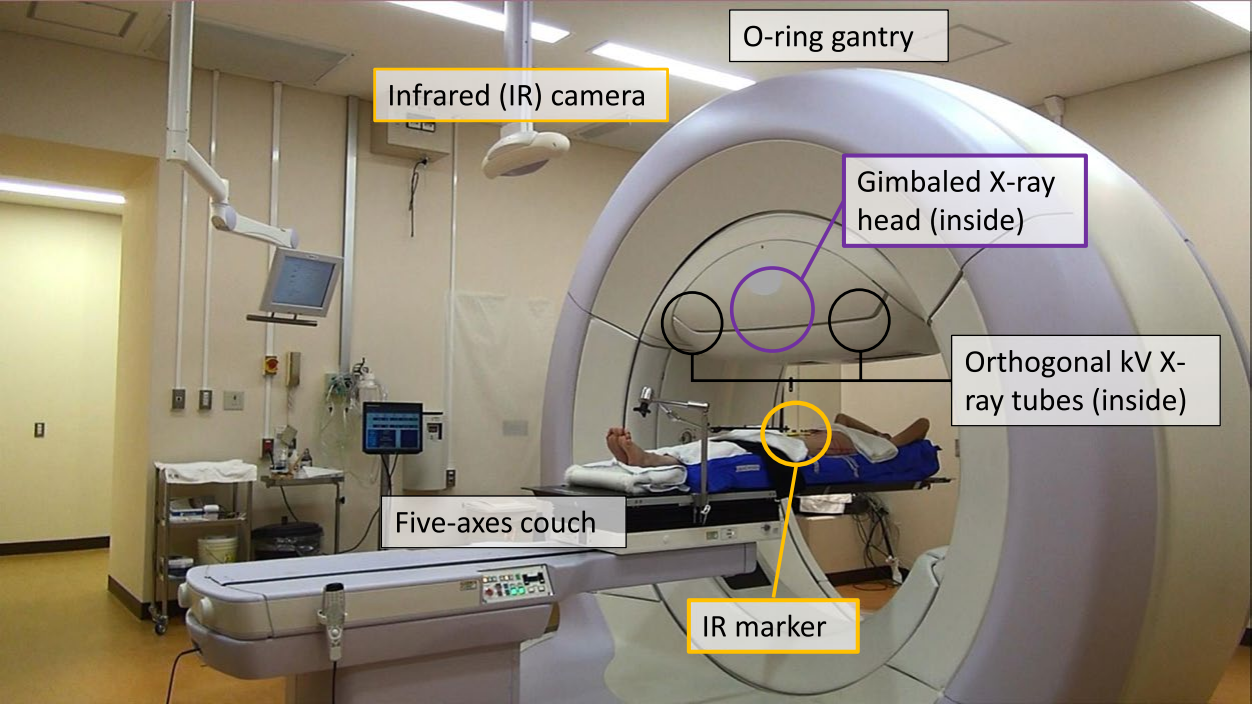
Overall structure of the Vero4DRT.

The Vero4DRT (Mitsubishi Heavy Industries Ltd, Japan and BrainLAB AG, Germany) is an innovative treatment machine that has been developed since 2002 under the initiative of Kyoto University with the aim of realizing the 4D-IMRT for moving tumors. Two specific features are incorporated into the Vero4DRT for this purpose. First, a pair of orthogonal kV X-ray imagers on the gantry can detect the real-time 3D tumor motion. Second, the gimbaled X-ray head can swing the intensity-modulated beams directly toward the moving tumor and can cover a field size of 15 × 15 cm, which is enough for the extent of LAPC. These features can effectively realize the dynamic tumor-tracking IMRT (DTT-IMRT) with real-time monitoring (Figs 1 and S1). Based on the fundamental assessments for 4DRT performance^20–27^, we started DTT-IMRT with Vero4DRT in June 2013, and reported the initial clinical outcomes^26^.

The objectives of this study were to report on the initial experience, the detailed analyses of tumor-tracking accuracy, and the practical benefits of DTT-IMRT for LAPC.

## Results

### Patient and treatment characteristics

From June 2013 to March 2015, a total of 10 patients were enrolled in this study. The patient and tumor characteristics and treatment details are summarized in Table 1. All patients had non-progressive and unresectable LAPC after three courses of induction chemotherapy. Visicoil fiducial marker (0.5 or 0.75 × 10 mm, IBA Dosimetry GmbH, Germany) insertion was performed percutaneously in three patients and endoscopically in seven patients with no obvious adverse events during and after the marker insertion. Patients were treated with 45–51 Gy in 15 fractions (a median of 48 Gy) without interruption in a median of 21 days. Concurrent chemotherapy included the weekly gemcitabine (1000 mg/m^2^) in eight patients, and daily S-1 (80 mg/m^2^, oral intake) in one patient. Gemcitabine administration was completed in six patients.

**Table 1:** Patient characteristics.

Characteristic		Value
Total		10
Gender	Female/Male	2/8
Age (years old)	Mean	71
	Range	64–79
PS	0/1	6/4
Tumor location	Head(uncus)/Body/Tail	5/5/0
Tumor size (mm)	Mean	20
	Range	20–50
Clinical Stage (UICC 7th)	Stage2A/2B/3	1/0/9
Treatment		
Radiotherapy dose	Median [Gy]	48
	45/48/51	1/5/4
Induction chemotherapy	Gemcitabine	9
	Gemcitabine and S-1	1
Concurrent chemotherapy	Gemcitabine	8
	S-1	1
Maintenance chemotherapy	Gemcitabine	8
	Gemcitabine and S-1	1
Surgical resection		0

*Abbreviations*: PS = performance status; UICC = The International Union Against Cancer; S-1 = a combined drug of tegafur, gimestat, and otastat potassium.

### Planning target volume size reduction and 4D dose distribution

The planning target volume (PTV) in DTT-IMRT was 18% smaller than that created with a conventional internal target volume (ITV) method. The mean of PTV in ITV method was 228 mL (range, 173–327 mL), while that in DTT-IMRT was 186 mL (range, 144–284 mL). There was no significant difference of PTV reduction between patients with head and body tumors (17% and 19% in head and body tumor, respectively). All IMRT plans adopted a 5-mm CTV-to-PTV margin because the patient-specific modelling errors estimated after the CT simulation were less than a 5-mm. All IMRT plans fulfilled the dose-volume constraints on the mid-ventilation CT. The difference of dose distribution on each phase of 4D-CT from the planned dose on the mid-phase CT was calculated, and the planned dose distribution was revealed to be well reproduced on 4D-CT. Mean (±SD) difference from the planned GTV D95% and CTV D95% values were +1.5% (±3.4%) and +1.3% (±2.9%), respectively. Mean difference from the planned stomach V45 and V42 were −0.23 mL (±0.38 mL) and −1.08 mL (±1.48 mL), respectively. The doses to the stomach were well under the dose constraints, and 97% and 99% of the 100 4D-CT phases (10 phases × 10 pts) met the constraints of V45 ≤ 1 mL and V42 ≤ 5 mL, respectively. The mean difference from the planned duodenum V45 and V42 were −0.13 mL (±1.60 mL) and −1.29 mL (±2.01 mL), respectively. The 99% and 99% of 4D-CT phases met the duodenum constraints of V45 ≤ 1 mL and V42 ≤ 5 mL, respectively. The dose to the other OARs were well under the dose constraints. Samples of dose distribution and dose-volume histogram are shown in Figs S2 and S3.

### Tumor motions

Mean ± SDs of peak-to-peak tumor motions on the simulation 4D-CT were 2.8 ± 1.8 mm, 2.9 ± 1.3 mm, and 7.6 ± 3.8 mm in the left-right (LR), anterior-posterior (AP), and cranial-caudal (CC) directions, respectively. Those in the head tumor were 2.7 mm, 2.7 mm, and 6.8 mm, respectively, and those in the body tumor were 2.8 mm, 3.1 mm, and 8.4 mm, respectively.

**Figure 2:**
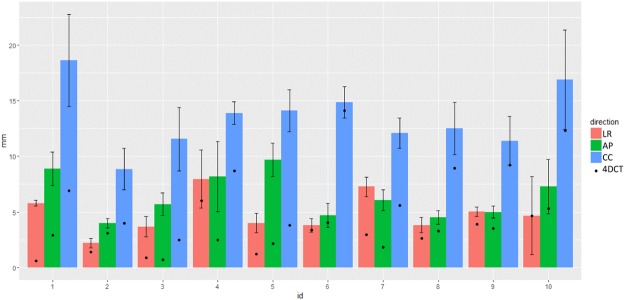
Peak-to-peak tumor motion in the simulation 4D-CT and the daily treatment. Each black dot represents the mean amount of peak-to-peak tumor motion in the simulation 4D-CT. Each solid bar and error bar indicates the mean peak-to-peak tumor motion and its standard deviation during the whole treatment.

A total of 46,320 monitoring kV-images over all patients and treatment fractions were exported from the Vero4DRT system and evaluated. Mean ± SDs of daily peak-to-peak tumor motions were significantly larger than those on simulation 4D-CT: those in LR, AP, and CC directions were 4.7 ± 2.3 mm, 6.2 ± 2.5 mm, and 13 ± 3.3 mm, respectively. Those in the head tumor were 5.4 mm, 6.3 mm, and 13.2 mm, respectively, while those in the body tumor were 4.2 mm, 6.5 mm, and 13.8 mm, respectively. The tumor movement in each patient is illustrated in Fig. 2.

Mean ± SDs of daily maximum velocity of tumor motions were 3.0 ± 0.7 mm/s, 3.8 ± 0.4 mm/s, and 10 ± 0.7 mm/s in LR, AP, and CC directions, respectively. Mean ± SDs of velocity in 3D radial motion was 3.8 ± 2.9 mm/s (range, 0.0–43 mm/s).

When an average of exhale peak positions in the first/last 20 s of each treatment day were defined as the first/last baseline tumor positions, mean ± SDs of the end-to-end position difference in CC direction were −0.7 ± 1.7 mm (range, −2.8–6.5 mm). During the daily treatments, the ≥5 mm deviations of exhale peak positions from the first baseline position were observed in 0.01%, 0.3%, 1.7%, and 3.3% in LR, AP, CC, and 3D directions, respectively. Similarly, the ≥2.5 mm deviations of exhale peak positions were observed in 2.1%, 3.5%, 11.1%, and 21.2% in LR, AP, CC, and 3D directions, respectively. To cover the exhale peak positional changes from the first baseline position, 2.6-mm, 2.8-mm, and 5.8-mm of margins in LR, AP, and CC directions, respectively, were calculated according to the Van Herk formula (PTVmargin = 2.5 Σ + 0.7 σ). Even with these margins, however, significant positional deviations by more than the estimated margin size were observed in AP direction in two cases; exhale peak positions were deviated by more than AP margins in 18% and 11% of cases #1 and #5, respectively. Table 2 summarizes the daily motions in each patient. Samples of tumor motion are shown in Fig. S4.

**Table 2:** Characteristics of daily tumor movement.

Pt.	Maximum velocity [mm/s]*			End-to-end [mm]*	Deviated peak positions [%]^†^		
	LR	AP	CC	CC	LR	AP	CC
1	2.5 ± 0.5 (2.1/3.4)	3.9 ± 1.0 (2.8/5.5)	10.4 ± 1.6 (8.3/11.9)	2.4 ± 2.9 (−0.7/5.2)	2.4	18.4	5.1
2	0.9 ± 1.2 (0.1/4.6)	2.1 ± 0.9 (1.0/4.7)	5.8 ± 0.8 (4.5/7.3)	−0.1 ± 0.9 (−1.9/1.1)	0.1	0.0	0.1
3	2.9 ± 2.1 (1.0/7.9)	4.8 ± 2.6 (1.6/9.7)	8.2 ± 1.9 (5.8/12.5)	1.3 ± 2.2 (−2.0/6.5)	3.1	0.3	2.1
4	4.5 ± 1.0 (3.2/7.1)	3.2 ± 1.1 (1.5/5.0)	11.6 ± 1.5 (9.2/14.5)	−0.5 ± 0.9 (−2.3/1.3)	0.6	0.3	0.5
5	1.5 ± 0.8 (0.4/3.0)	5.3 ± 0.9 (3.8/6.9)	9.7 ± 2.5 (6.6/17.9)	1.7 ± 2.3 (−1.6/6.1)	3.8	11.0	3.1
6	2.4 ± 1.2 (0.9/5.3)	2.9 ± 1.4 (1.3/7.2)	12.3 ± 1.8 (10.1/15.2)	0.3 ± 1.8 (−2.8/4.7)	1.6	0.1	0.7
7	5.9 ± 2.9 (2.8/13.6)	4.3 ± 1.8 (2.2/8.3)	8.9 ± 1.9 (7.0/12.9)	1.1 ± 1.3 (−0.8/3.3)	2.3	1.8	0.9
8	2.0 ± 0.9 (0.5/3.7)	3.9 ± 2.5 (1.6/11.0)	11.1 ± 2.3 (8.2/15.9)	0.1 ± 1.0 (−2.0/1.6)	2.1	0.0	0.0
9	3.7 ± 0.6 (2.5/4.9)	2.7 ± 0.5 (2.0/3.8)	9.1 ± 1.6 (6.7/11.7)	0.8 ± 0.7 (−0.7/1.9)	1.1	0.3	0.0
10	2.7 ± 3.1 (0.6/12.6)	4.9 ± 1.6 (3.2/9.9)	14.1 ± 2.2 (11.6/18.3)	0.6 ± 1.3 (−1.8/3.2)	1.8	0.5	0.2

*All data are shown in mean or mean (±SD) values (and minimum/maximum values) of daily treatment fractions. ^†^The proportion of exhale peak tumor positions which deviated from the daily baseline position by more than estimated margin sizes (2.6 mm, 2.8 mm, and 5.8 mm in LR, AP, and CC directions) is shown.

*Abbreviations:* LR = left to right; AP = anterior to posterior; CC = cranial to caudal.

### Tracking error analyses

Mean ± SDs of the geometric errors between the predicted and detected marker were evaluated and revealed to be considerably small: 0.6 ± 1.2 mm, −0.1 ± 1.4 mm, and 0.6 ± 1.4 mm in LR, AP and CC directions, respectively (Table 3). The root-mean-squared errors and the 95^th^ percentile of absolute errors were 1.3 and 2.7 mm, 1.2 and 2.4 mm, and 1.5 and 2.9 mm in LR, AP, and CC directions, respectively. The geometric errors of ≥5 mm were observed only for 0.09%, 0.11%, and 0.18% in LR, AP, and CC directions, respectively. The overall mean and the 95^th^ percentile 3D radial errors were 2.1 and 3.9 mm, respectively.

**Table 3:** Geometric errors for daily treatment.

Pt.	Geometric error [mm]			Absolute geometric error [mm]		
	LR	AP	CC	LR	AP	CC
1	0.3 ± 1.0 (−3.0/4.3)	0.3 ± 1.6 (−4.2/5.9)	0.2 ± 2.2 (−6.7/5.9)	0.8 ± 0.7 (0/4.3)	1.2 ± 1.0 (0/5.9)	1.9 ± 1.1 (0/6.7)
2	0.2 ± 0.8 (−2.1/6.7)	−0.4 ± 0.9 (−6.3/2.7)	0.9 ± 1.4 (−6.6/6.5)	0.6 ± 0.6 (0/6.7)	0.8 ± 0.6 (0/6.3)	1.4 ± 1.0 (0/6.6)
3	0.1 ± 1.2 (−10.9/23.6)	0.4 ± 1.7 (−15.1/32.1)	−0.3 ± 1.6 (−16.4/15.8)	0.8 ± 0.9 (0/23.6)	1.3 ± 1.2 (0/32.1)	1.2 ± 1.0 (0/16.4)
4	0.1 ± 1.0 (−8.8/9.3)	−1.1 ± 1.3 (−14.2/9.3)	1.3 ± 1.2 (−8.0/17.9)	0.8 ± 0.7 (0/9.3)	1.3 ± 1.0 (0/14.2)	1.5 ± 1.0 (0/17.9)
5	1.0 ± 1.5 (−3.0/5.2)	0.0 ± 1.4 (−3.6/9.7)	−0.1 ± 1.9 (−14.5/5.1)	1.5 ± 1.0 (0/5.2)	1.1 ± 0.8 (0/9.7)	1.5 ± 1.1 (0/14.5)
6	0.7 ± 1.1 (−9.1/5.2)	0.3 ± 1.1 (−8.2/4.2)	0.4 ± 1.2 (−5.7/5.8)	1.0 ± 0.8 (0/9.1)	0.9 ± 0.8 (0/8.2)	1.0 ± 0.8 (0/5.8)
7	0.5 ± 1.2 (−3.8/9.5)	−0.3 ± 1.0 (−3.7/10.3)	0.4 ± 1.2 (−9.6/3.8)	1.0 ± 0.8 (0/9.5)	0.8 ± 0.7 (0/10.3)	1.0 ± 0.7 (0/9.6)
8	1.6 ± 1.1 (−1.8/6.7)	−0.4 ± 1.0 (−4.5/6.9)	1.3 ± 1.3 (−4.9/5.9)	1.7 ± 1.0 (0/6.7)	0.8 ± 0.7 (0/6.9)	1.5 ± 0.9 (0/5.9)
9	0.1 ± 0.9 (−9.0/2.7)	0.1 ± 0.7 (−2.1/2.5)	0.5 ± 0.8 (−4.4/3.3)	0.7 ± 0.6 (0/9.0)	0.5 ± 0.4 (0/2.5)	0.8 ± 0.6 (0/4.4)
10	0.7 ± 1.2 (−5.0/14.7)	0.5 ± 0.9 (−5.4/15.2)	0.7 ± 1.0 (−17.2/8.9)	1.1 ± 0.7 (0/14.7)	0.8 ± 0.6 (0/15.2)	1.0 ± 0.8 (0/17.2)

All data are shown in mean or mean ± SD values (and minimum/maximum values) of daily treatment fractions.

*Abbreviations:* LR = left to right; AP = anterior to posterior; CC = cranial to caudal.

### Time aspects of DTT-IMRT workflow

The mean (±SD) total monitoring time (including DTT-IMRT delivery) per fraction was 5.8 min (±0.1 min). The mean in-room time was 24.5 min.

### Clinical outcomes

With a median follow-up period of 25.9 months, the 1-year and 2-year overall survival (OS) from the induction chemotherapy were 100% and 50%, respectively. The median overall survival was 25.9 months (95% confidence interval (CI) 17.4–not available). The 2-year and median progression-free survival (PFS) from the induction chemotherapy were 30% and 14.6 months (95% CI 7.7–not available), respectively. The 2-year local control rate was 74%. During DTT-IMRT with gemcitabine, G3 leucopenia and G3 neutropenia were observed in two and one each. Two patients had G2 GI-toxicity including anorexia and nausea. At late phase, one patient developed G3 anemia due to gastric antral vascular ectasia and received several blood transfusions and endoscopic intervention^26^.

## Discussion

The present study aimed to report on the first experiences of DTT-IMRT with the Vero4DRT to the moving target of LAPC under patients’ voluntary breathing. Among real-time adaptation systems^2–7^, the Vero4DRT system confers definite advantages in DTT-IMRT. First, a treatment field large enough to cover LAPC facilitates the accurate dose delivery within a reasonable treatment time. Second, a short interval of the monitoring imaging frequency is applicable, which is necessary for the prompt detection of internal/external correlation error. Third, the latency with Vero4DRT is the smallest recorded (close to a 50 ms), which contributes to the overall tumor tracking and treatment accuracy. To recognize the benefits of this new treatment platform of DTT-IMRT, we focused on reporting the tumor-tracking accuracy and the practical aspects of DTT-IMRT for the treatment of LAPC.

In the current study, we estimated that the PTV size in 4DRT was reduced by 18% compared with the conventional ITV-based PTV. This PTV reduction itself may be beneficial to patients in terms of GI toxicities. Furthermore, the benefits of our DTT-IMRT are pronounced on the accuracy and reliability in delivering intensity-modulated beams. This is because the dose-limiting GI OARs are serial organs and suffer from a high-dose to small volume^28,29^, which can theoretically appear due to the dose uncertainties in IMRT for moving tumor. The accuracy of our DTT-IMRT system is supported by the smallest tumor-tracking errors, in which the presented 95^th^ percentile geometric errors were 2.7 mm, 2.4 mm, and 2.9 mm in LR, AP, and CC directions, respectively. This accuracy is as high as the robotic tracking system for upper abdominal tumor, in which Winter *et al*. reported the 95^th^ percentile errors of 2.1 mm, 1.8 mm, and 3.3 mm in LR, AP, and CC directions, respectively, during stereotactic body radiotherapy (SBRT) in 27 patients with liver cancer radiotherapy^30^. Overall, we concluded it was acceptable to use a 5-mm expansion for CTV-to-PTV margin in DTT-IMRT for LAPC. This margin size contributes to the reduction of PTV, and is also beneficial to patients compared with a 1–2 cm expansion recommended when daily image guidance is unavailable^16,31^.

The investigation of velocity of LAPC motion was unique in the present study (Table 2); the 3D radial motion of 3.8 mm/s was smaller than that in lung and liver tumors, and well within the maximum velocity of 152 mm/s in the gimbaled head motion at isocenter level. The averaged in-room treatment time of 24.5 min is relatively shorter than that in previous lung and liver SBRT studies (34.4–36 min)^4,24^, likely because the number of fractions is larger so that the daily dose (3.0–3.4 Gy) and the monitor units are smaller despite the step-and-shoot IMRT delivery.

The conventional respiratory-managed IMRT is currently an important treatment option for LAPC, but is subjected to underlying uncertainties; we presented the discrepancy of tumor motions between the simulation 4D-CT and the daily treatment (Fig. 2) which agrees with previous reports^31–36^. Additionally, we showed that a variety of the intrafractional baseline drift of tumor positions appeared in all 10 patients (Table 2 and Fig. S4). The estimated margins with van Herk formula to compensate the baseline drift of exhale peak tumor position were significantly large in CC direction, but obviously insufficient in AP direction in two patients who had relatively larger baseline drifts among participants. This implicates a difficulty of estimating population-based margins for respiratory-gating, and poses some challenges to the accurate delivery of IMRT with the respiratory gating strategy^37^. Also, recent reports revealed the residual tumor motions during breath holding with 2.9 mm (superior) and 3.8 mm (inferior), or up to 11.0 mm in superior-inferior direction^17,18^. This level should be nontrivial when breath-hold IMRT delivers an aggressive dose with steep dose gradients or a hypofractionated dose regimen. The presented uncertainties in tumor positions together with these previous studies highlight the benefits of DTT-IMRT for LAPC. Because these uncertainties cannot be reflected on a single simulation CT/4D-CT, we did not focus on the dosimetric advantages of DTT-IMRT over the respiratory-managed IMRT.

One key concern in IMRT delivery for moving tumors is the possibility of interplay between tumor motion and multi-leaf collimator (MLC) motion. Reportedly, large dose variations can appear in a single fraction in IMRT for moving tumors^38^, and we also observed this phenomenon in the quality assurance (QA) process by a moving phantom; the γ index passing rates with the criterion of 3%/1 mm was 69.8% without tracking^39^. Although a series of previous studies demonstrated that dosimetric errors were cancelled out through multiple fractions^40–42^, we support the necessity of considering these errors because large intra- and interfractional motion variations in LAPC are observed and it is obscure whether the errors are properly cancelled under such a motion. The dosimetric accuracy in a single fraction of our DTT-IMRT is high (the γ index passing rates, 92.9% ± 4.0%)^39^, which indicates that DTT-IMRT can minimize the dosimetric errors from the interplay and realize a reliable IMRT delivery for moving targets.

The uncertainty due to internal-external correlation change is also a relevant issue in 4DRT with IR tracking technique^25^. The internal-external correlation model is vulnerable to the changes of motion patterns of internal tumor or external IR marker. As described above, there are a variety of the baseline drift of tumor positions, which is one example of introducing the changes of tumor motion patterns. In our Vero4DRT system, we adopted an imaging interval of every 1 second and could detect large or continuous deviations of the predicted marker from the tolerance circle. This real-time visualization system enhances the reliability of DTT-IMRT delivery, and contributed to the reduction of geometric errors.

We demonstrated the tumor tracking and dosimetric accuracy during DTT-IMRT but, however, we admit there are also assumptions in this study. First, we did not investigate if the implanted marker is an adequate surrogate for the tumor position by using a daily soft tissue imaging, such as cone beam CT (CBCT). Although Van der Horst *et al*. showed that there is no migration of fiducials with time^43^ and we also confirmed it with the follow-up CT images after DTT-IMRT (data not shown), a daily soft tissue imaging should be ideally used to assess the target and marker positions during treatment course. Similarly, we did not visualize the location of OARs with respect to the target during the treatment. We demonstrated that the dose distribution on each phase of the simulation 4DCT is well acceptable, but the delivered dose to luminal OARs can become obscure when their position and volume change greatly, as we reported previously^44^. Now that Vero4DRT system is equipped with 4D-CBCT function, future studies with 4D-CBCT prior or after daily treatment will shed light on the adequacy of the marker and target position as well as the interfractional dosimetric changes in OARs during DTT-IMRT treatment course.

Recently, the image-guided RT arsenal was updated to include a new real-time soft tissue imaging technique, such as the magnetic resonance image (MRI)-guided RT^45^. Although both Vero4DRT and MRI-RT systems can address the issues in IMRT for moving tumors, the major advantage of Vero4DRT is the aforementioned ability to swing intensity-modulated beam toward tumor and it does not require patients’ contribution for breath-holding to achieve an accurate or efficient tumor tracking. This results in the reduced in-room treatment time and the minimized patient burden, especially in elderly patients or those who feel uncomfortable in controlling respiration. In addition, Vero4DRT does not have contraindications, including a pacemaker and non-MR compatible implants. The drawback of Vero4DRT is that it requires additional radiation exposure and an inserted fiducial marker. Further, the movement of OARs during DTT-IMRT cannot be assessed in Vero4DRT. Overall, both Vero4DRT and MRI-RT systems have demonstrated high accuracy in real-time tumor tracking^45^. These innovations in 4DRT have a strong potential to improve the clinical outcomes and should be further investigated in clinical studies.

DTT-IMRT with Vero4DRT is applicable to cancers that satisfy two key features: the tumor should be accessible to a fiducial marker, and the treatment field should be within 15 × 15 cm. As presented in the current study, our first target was LAPC. Although the evaluation of clinical outcomes was beyond the scope of this study, we observed a promising local control rate (the 2-y local control rate of 74%) with a median survival of 25.9 months at a median follow-up period of 25.9 months. These promising outcomes inspired our inclination to further use the advantage of DTT-IMRT, and we started an early phase II multi-institutional study in 2015 to test the feasibility of DTT-IMRT for LAPC (UMIN000017521).

In summary, the clinical application of DTT-IMRT for LAPC patients was investigated. The 18% reduction of PTV with DTT-IMRT was observed compared with the conventional ITV-based approach. The dose distribution on a representative CT is recalculated on each of 4D-CT phase to confirm the target coverage and OAR dose sparing during DTT-IMRT. The QA process confirmed that DTT-IMRT minimized the dosimetric errors from the interplay between tumor motion and MLC motion. The detailed analyses on daily tumor motions and tumor-tracking errors revealed that the 95^th^ percentiles of tumor-tracking errors were 2.4–2.9 mm despite a variety of the intrafractional and interfractional tumor motions in all 10 patients. The in-room treatment time was 24.5 min on average. The preliminary results on local tumor control and toxicity were promising, and an early phase II multi-institutional study was established to investigate the feasibility of DTT-IMRT for LAPC.

## Methods

### Patients eligibility criteria

This was a single-institution study, approved by the Institutional Review Board (Ethics Committee Graduate School and Faculty of Medicine Kyoto University, KUHP-E1675) in compliance with the Helsinki Declaration. The primary endpoint of this study was to evaluate the accuracy of DTT-IMRT for abdominal cancer, and the eligibility criteria included patients with clinical stage II–III of unresectable LAPC who received neither curative-intent surgery nor radiotherapy, had pathological confirmation of adenocarcinoma, and provided written informed consent.

### CT simulation

One Visicoil was implanted inside the tumor. At least 1 week after the insertion, patients were immobilized in the BodyFix system (Medical Intelligence, Germany) with both arms raised overhead. After ≥3 hours of fasting time, patients were examined with contrast-enhanced computed tomography (CT) under free breathing using a LightSpeed RT 16-slice CT simulator (GE Healthcare, UK) and a real-time positioning management system (Varian Medical Systems, USA). The periodic whole images were sorted into 10 phased bins of 4D-CT images using the Advantage Workstation (GE Healthcare, UK). Following the CT simulation, 4D modeling was performed on Vero4DRT to assess the patient-specific modelling error.

### Treatment planning

The gross tumor volume (GTV) included the tumor and metastatic lymph nodes. The clinical target volume (CTV) was defined as GTV plus a 5-mm margin as well as the retropancreatic space and the paraaortic lymph nodes between the celiac axis and the superior mesenteric artery. These target volumes and the luminal OARs (the stomach and the duodenum) were delineated on all 10 phases of 4D-CT and were overlaid onto a mid-ventilation phase. The CTV-to-PTV margin was defined as the sum of a setup error (1.0 mm) and additional margins, which compensate the 4D modelling error (mean + 2 SD mm), baseline drift of abdominal position, and mechanical errors (<0.5 mm)^27^. When the sum of these margins exceeds 5 mm, the CTV-to-PTV margin size was defined as 5 mm. The stomach and the duodenum were regarded as critical OARs for severe gastrointestinal (GI) toxicities^28,29^. To mitigate the interfractional dose changes, the planning organs-at-risk volume was created with 3- to 7-mm margin sizes at the discretion of the treating physician^44,46^. Other OARs, including the bowels, the liver, the kidneys, and the spinal cord, were also delineated on the mid-ventilation phase.

The IMRT treatment planning was performed on the iPlan RT Dose (BrainLab, Germany). Six coplanar or non-coplanar 6-MV photon beams (80°–280° in every 40°) were used and the anterior beams were avoided because the depth-dose distribution was influenced by the respiratory movement of abdominal wall. The prescription dose was specified to D95 (the dose that covers 95% of the structure) of PTV-boost, a volume that subtracted the planning organs-at-risk from PTV. The choice of prescription dose was individualized between 45 and 51 Gy/15 fr. by achieving the dose constraints for OARs. The whole PTV was kept over 36 Gy according to consensus guidelines^10^. The dose constraints for OARs were as follows: V20 (volume receiving ≥20 Gy) of at least ipsilateral kidney ≤30%; the mean dose of liver ≤30 Gy; the maximum dose to spinal cord <36 Gy; the V42 and V45 of the integrated luminal OARs (stomach or duodenum) ≤5 mL and ≤1 mL, respectively. The V45 was determined referring to previous reports; 55 Gy/28–30 fr. to 1–2 mL of stomach or duodenum significantly associated with severe GI toxicities^28,29^ and this dose/fraction approximates 45 Gy/15 fr. with regards to the late effects. The dose constraints are summarized in Table 4. The monitor units were calculated with the X-ray voxel Monte Carlo algorithm, with a spatial resolution of 2 mm and a variance of 2%.

**Table 4:** Dose-volume constraints for the IMRT plan.

Structure	Constraints
PTV-boost	D95% = 100%*
PTV	D98% > 36 Gy^†^
Stomach, Duodenum	V45Gy < 1.0 mL^‡^
	V42Gy < 5.0 mL
	V39Gy < 25.0 mL
POV (each)	V39Gy < 30.0 mL
	V36Gy < 45.0 mL
Kidney (each)	V20Gy < 30%
Liver	D_mean_ < 30 Gy
Spinal Cord	D_max_ < 36 Gy

*Abbreviations:* Dxx% = the dose covering the ≥xx% of the structure volume; D_max_ = maximum dose; D_mean_ = mean dose; VxxGy = the volume of the structure receiving >xx Gy; PTV-boost = PTV minus POV structures; POV = each planning organs-at-risk structure for the stomach or duodenum.

^*^The dose relative to the prescription dose.

^†^The whole PTV was kept over 36 Gy according to consensus guidelines^10^.

^‡^The V45 was determined referring to previous reports; 55 Gy/28–30 fr. to 1–2 mL of stomach or duodenum significantly associated with severe GI toxicities^28,29^ and this dose/fraction approximates 45 Gy/15 fr. with regards to the late effects.

### 4D dose calculation and Quality assurance

To assess the dose distribution on each phase of 4D-CT, the monitor units and leaf motion were imported from iPlan and were used to simulate step-and-shoot IMRT with in-house developed Monte-Carlo simulation, as described previously^47^. A four-axes moving phantom was used to assess the differences between the planned dose-distribution and the delivered doses during static and moving conditions. This QA system and detailed procedure were described previously^39^.

### Dynamic tumor-tracking IMRT procedure

**Figure 3:**
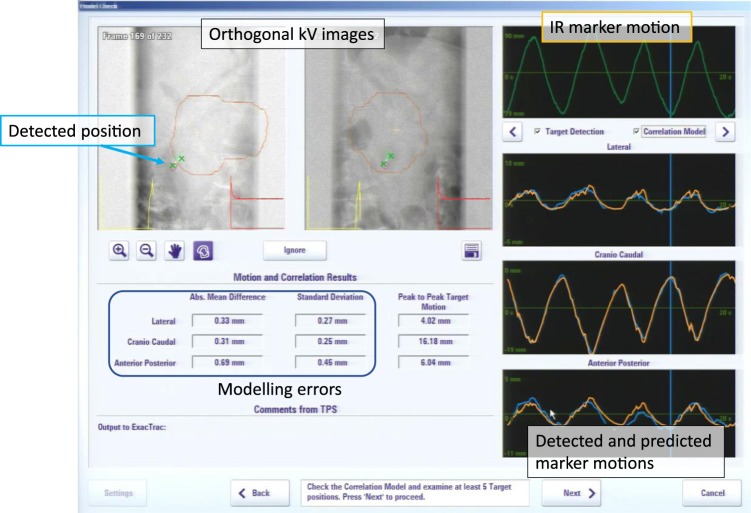
4D modeling. During 4D modelling, the fluoroscopic kV images (upper left) are acquired from the orthogonal kV imagers on the gantry, and both ends of the linear fiducial marker inside the tumor are automatically detected. Simultaneously, the position and motion of the infrared (IR) marker on the patient’s abdomen is captured (green curve in the upper right column). The correlation model between the internal marker and the IR marker motion is generated, and then the marker motion and the predicted marker motion are depicted on the right lower columns in three directions. Finally, the mean and SDs of the differences between the detected marker position and the predicted position are calculated and shown in lower left column (modeling errors).

**Figure 4:**
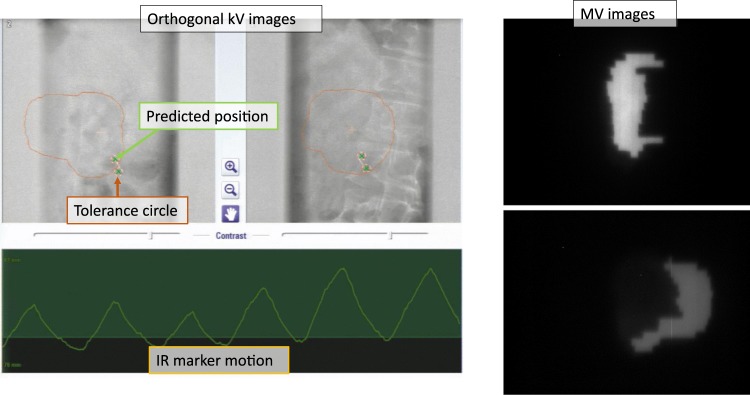
kV and MV X-ray monitoring images during DTT-IMRT. During DT-IMRT beam delivery, the predicted position of the internal fiducial marker (green x) and tolerance circle (a 3-mm radius) are superimposed on the orthogonal fluoroscopic kV images. The kV images are updated every 1 sec. When the predicted positions deviate from the actual fiducial marker positions, observers can consider the recalibration of the 4D model.

DTT-IMRT consists of the following four steps: (1) Patients are immobilized in BodyFix and the bony-based setup error correction is performed. (2) A 4D model is built immediately before the daily treatment. A pair of gantry-mounted orthogonal X-ray imagers and an infrared (IR) (ExacTrac, BrainLAB AG, Germany) marker on the upper abdomen (Fig. 1) are utilized to generate a correlation 4D model between the internal target and external surface of the patient’s abdomen (Fig. 3). Once established, the correlation model allows Vero4DRT to predict the future target position based on the position and movement of the IR marker. (3) The gimbaled head swings the intensity-modulated beams to follow the target trajectory in real-time under free breathing using the 4D model and IR marker. During DTT-IMRT, the Visicoil is monitored every 1 second with kV and MV X-ray imagers. The predicted marker positions are superimposed on the kV images and are surrounded by a 3-mm radius of tolerance circle (Fig. 4). (4) The 4D model is rebuilt when the operator observes that the marker has deviated from the predicted position and is systematically outside of the tolerance circle.

### Evaluation

The following factors were evaluated. (1) The volume reduction of PTV between DTT-IMRT and the conventional ITV method, which creates PTV by adding a 5-mm margin to the sum of CTVs on all 4D-CT, was evaluated. (2) The dose distribution on each phase of 4D-CT was evaluated. (3) The differences between the tumor movement on the simulation 4D-CT and that on daily treatments were studied. The peak-to-peak, velocity, and the baseline exhale tumor positions were analyzed. Additionally, the margin sizes to compensate the change of baseline exhale peak positions were estimated according to the Van Herk formula. (4) The geometric errors between the predicted and the detected positions were evaluated. (5) The in-room time for the daily treatment was measured. Clinical outcomes were also evaluated, including OS, PFS, and the toxicity. The survival outcomes were calculated from day 1 of the induction chemotherapy and were estimated by Kaplan-Meier method. Toxicity was graded according to the NCI Common Terminology Criteria for Adverse Events, version 4.0.

### Ethics approval and consent to participate

This study was approved by the Institutional Review Board in Kyoto University Hospital. (reference number: KUHP-E1675).

## Electronic supplementary material

Fig S1-S4

## Data Availability

The datasets used during the current study are available from the authors on reasonable request.

## References

[1] Keall PJ (2006). The management of respiratory motion in radiation oncology report of AAPM Task Group 76. Med. Phys..

[2] Koong AC (2004). Phase I study of stereotactic radiosurgery in patients with locally advanced pancreatic cancer. Int. J. Radiat. Oncol. Biol. Phys..

[3] Kamino Y (2006). Development of a four-dimensional image-guided radiotherapy system with a gimbaled X-ray head. Int. J. Radiat. Oncol. Biol. Phys..

[4] Matsuo Y (2014). Evaluation of dynamic tumour tracking radiotherapy with real-time monitoring for lung tumours using a gimbal mounted linac. Radiother. Oncol..

[5] Keall PJ (2014). The first clinical implementation of electromagnetic transponder-guided MLC tracking. Med. Phys..

[6] Booth JT (2016). The first patient treatment of electromagnetic-guided real time adaptive radiotherapy using MLC tracking for lung SABR. Radiother. Oncol..

[7] D’Souza WD, Naqvi SA, Yu CX (2005). Real-time intra-fraction-motion tracking using the treatment couch: a feasibility study. Phys. Med. Biol..

[8] Siegel RL, Miller KD, Jemal A (2016). Cancer statistics, 2016. CA Cancer. J. Clin..

[9] Hammel P (2016). Effect of chemoradiotherapy vs chemotherapy on survival in patients with locally advanced pancreatic cancer controlled after 4 months of gemcitabine with or without erlotinib: the LAP07 randomized clinical trial. JAMA..

[10] National Comprehensive Cancer Network. NCCN clinical practice guidelines in oncology. Pancreatic Adenocarcinoma Version 2.2018, http://www.nccn.org/professionals/physician_gls/pdf/pancreatic.pdf (2018)

[11] Bittner MI, Grosu AL, Brunner TB (2015). Comparison of toxicity after IMRT and 3D-conformal radiotherapy for patients with pancreatic cancer - a systematic review. Radiother. Oncol..

[12] Ben-Josef E (2012). A phase I/II trial of intensity modulated radiation (IMRT) dose escalation with concurrent fixed-dose rate gemcitabine (FDR-G) in patients with unresectable pancreatic cancer. Int. J. Radiat. Oncol. Biol. Phys..

[13] Passoni P (2013). Hypofractionated image-guided IMRT in advanced pancreatic cancer with simultaneous integrated boost to infiltrated vessels concomitant with capecitabine: a phase I study. Int. J. Radiat. Oncol. Biol. Phys..

[14] Feng M (2009). Characterization of pancreatic tumor motion using cine MRI: surrogates for tumor position should be used with caution. Int. J. Radiat. Oncol. Biol. Phys..

[15] Gwynne S (2009). Respiratory movement of upper abdominal organs and its effect on radiotherapy planning in pancreatic cancer. Clin. Oncol. (R. Coll. Radiol.).

[16] Huguet F, Goodman KA, Azria D, Racadot S, Abrams RA (2012). Radiotherapy technical considerations in the management of locally advanced pancreatic cancer: American-French consensus recommendations. Int. J. Radiat. Oncol. Biol. Phys..

[17] Shinohara ET (2012). Feasibility of electromagnetic transponder use to monitor inter- and intrafractional motion in locally advanced pancreatic cancer patients. Int. J. Radiat. Oncol. Biol. Phys..

[18] Lens E, van der Horst A, Versteijne E, Bel A, van Tienhoven G (2016). Considerable pancreatic tumor motion during breath-holding. Acta. Oncol..

[19] Malinowski K, McAvoy TJ, George R, Dietrich S, D’Souza WD (2012). Incidence of changes in respiration-induced tumor motion and its relationship with respiratory surrogates during individual treatment fractions. Int. J. Radiat. Oncol. Biol. Phys..

[20] Nakamura M (2010). Dosimetric characterization of a multileaf collimator for a new four-dimensional image-guided radiotherapy system with a gimbaled x-ray head, MHI-TM2000. Med. Phys..

[21] Depuydt T (2011). Geometric accuracy of a novel gimbals based radiation therapy tumor tracking system. Radiother. Oncol..

[22] Miyabe Y (2011). Positioning accuracy of a new image-guided radiotherapy system. Med. Phys..

[23] Mukumoto N (2013). Accuracy verification of infrared marker-based dynamic tumor-tracking irradiation using the gimbaled x-ray head of the Vero4DRT (MHI-TM2000). Med. Phys..

[24] Depuydt T (2013). Initial assessment of tumor tracking with a gimbaled linac system in clinical circumstances: a patient simulation study. Radiother. Oncol..

[25] Akimoto M (2013). Predictive uncertainty in infrared marker-based dynamic tumor tracking with Vero4DRT. Med. Phys..

[26] Goto Y (2018). Clinical results of dynamic tumor tracking intensity-modulated radiotherapy with real-time monitoring for pancreatic cancers using a gimbal mounted linac. Oncotarget..

[27] Mukumoto N (2014). Intrafractional tracking accuracy in infrared marker-based hybrid dynamic tumour-tracking irradiation with a gimballed linac. Radiother. Oncol..

[28] Nakamura A (2012). Analysis of dosimetric parameters associated with acute gastrointestinal toxicity and upper gastrointestinal bleeding in locally advanced pancreatic cancer patients treated with gemcitabine-based concurrent chemoradiotherapy. Int. J. Radiat. Oncol. Biol. Phys..

[29] Kelly P (2013). Duodenal toxicity after fractionated chemoradiation for unresectable pancreatic cancer. Int. J. Radiat. Oncol. Biol. Phys..

[30] Winter JD, Wong R, Swaminath A, Chow T (2015). Accuracy of robotic radiosurgical liver treatment throughout the respiratory cycle. Int. J. Radiat. Oncol. Biol. Phys..

[31] Reese AS, Lu W, Regine WF (2014). Utilization of intensity-modulated radiation therapy and image-guided radiation therapy in pancreatic cancer: is it beneficial?. Semin. Radiat. Oncol..

[32] Minn AY (2009). Pancreatic tumor motion on a single planning 4D-CT does not correlate with intrafraction tumor motion during treatment. Am. J. Clin. Oncol..

[33] Ge J, Santanam L, Noel C, Parikh PJ (2013). Planning 4-dimensional computed tomography (4DCT) cannot adequately represent daily intrafractional motion of abdominal tumors. Int. J. Radiat. Oncol. Biol. Phys..

[34] Jayachandran P (2010). Interfractional uncertainty in the treatment of pancreatic cancer with radiation. Int. J. Radiat. Oncol. Biol. Phys..

[35] Zhang H, Zhao G, Djajaputra D, Xie Y (2014). Determination of acquisition frequency for intrafractional motion of pancreas in CyberKnife radiotherapy. ScientificWorldJournal..

[36] Lischalk JW (2015). Four-dimensional computed tomography prediction of inter- and intrafractional upper gastrointestinal tumor motion during fractionated stereotactic body radiation therapy. Pract. Radiat. Oncol..

[37] Pierce G, Wang K, Gaede S, Battista J, Lee TY (2010). The effect of an inconsistent breathing amplitude on the relationship between an external marker and internal lung deformation in a porcine model. Med. Phys..

[38] Yu CX, Jaffray DA, Wong JW (1998). The effects of intra-fraction organ motion on the delivery of dynamic intensity modulation. Phys. Med. Biol..

[39] Mukumoto N (2016). Development of a four-axis moving phantom for patient-specific QA of surrogate signal-based tracking IMRT. Med. Phys..

[40] Jiang SB (2003). An experimental investigation on intra-fractional organ motion effects in lung IMRT treatments. Phys. Med. Biol..

[41] Bortfeld T, Jiang SB, Rietzel E (2004). Effects of motion on the total dose distribution. Semin. Radiat. Oncol..

[42] Schaefer M (2004). Influence of intrafractional breathing movement in step-and-shoot IMRT. Phys. Med. Biol..

[43] van der Horst A (2013). Interfractional position variation of pancreatic tumors quantified using intratumoral fiducial markers and daily cone beam computed tomography. Int. J. Radiat. Oncol. Biol. Phys..

[44] Nakamura A (2013). Interfractional dose variations in the stomach and the bowels during breathhold intensity-modulated radiotherapy for pancreatic cancer: Implications for a dose-escalation strategy. Med. Phys..

[45] Van Sörnsen de Koste, J. R. *et al*. MR-guided gated stereotactic radiation therapy delivery for lung, adrenal, and pancreatic tumors: a geometric analysis. *Int. J. Radiat. Oncol. Biol. Phys*. 2018 May 29. 10.1016/j.ijrobp.2018.05.048 30061007

[46] Crane CH (2016). Hypofractionated ablative radiotherapy for locally advanced pancreatic cancer. J. Radiat. Res..

[47] Ishihara Y (2014). Development of a dose verification system for Vero4DRT using Monte Carlo method. J. Appl. Clin. Med. Phys..

