# Supplementary material for: Evaluation of Dynamic Tumor-tracking Intensity-modulated Radiotherapy for Locally Advanced Pancreatic Cancer
Source: Sci Rep. 2018 Nov 20;8:17096. doi: 10.1038/s41598-018-35402-7 (PMC6244273; doi:10.1038/s41598-018-35402-7)
Supplement: Supplementary file 1 — Fig S1-S4 [file 41598_2018_35402_MOESM1_ESM.pdf]

Supplementary Figures

Article Title

Evaluation of Dynamic Tumor-tracking Intensity-modulated Radiotherapy for Locally Advanced Pancreatic Cancer

Authors:

Akira Nakamura, Masahiro Hiraoka, Satoshi Itasaka, Mitsuhiro Nakamura, Mami Akimoto, Yoshitomo Ishihara, Nobutaka Mukumoto, Yoko Goto, Takahiro Kishi, Michio Yoshimura, Yukinori Matsuo, Shinsuke Yano, and Takashi Mizowaki.

Supplementary Figure Legends

Fig. S1: Dynamic tumor-tracking IMRT.

A pair of gantry-mounted orthogonal kV X-ray imagers and an infrared marker on the abdomen are used to generate a correlation 4D model between the 3D position of internal target and external surface of the patient's abdomen (left). The gimbaled X-ray head can swing the intensity-modulated beam toward the tumor using the 4D model and the abdominal movement (right).

Fig. S2: Sample 4D dose distribution on the inhalation and exhalation phases of 4DCT.

Fig. S3: Samples of DVHs on 4D-CT.

4D dose distribution is calculated with the Monte-Carlo simulation method. The monitor units and leaf motions are exported from the treatment planning system. The delivery of an intensity-modulated beam is calculated on each 4D-CT. The dose volume histogram from 10 phases of 4D-CT is overlaid. The mean value of each structure is shown in thick red line, and each line represents the DVH on each 4D-CT image. The DVHs of GTV and CTV (top left), stomach (bottom left), and duodenum (bottom right) are shown.

Fig. S4: Samples of tumor movement.

The tumor movement (colored lines) in cranio-caudal direction throughout the treatment course for two patients (case #7 and #9) are shown. The different color of the tumor represents the different treatment day. Intra- and inter-fractional changes of tumor position and patterns of movement are observed.

Fig. S1

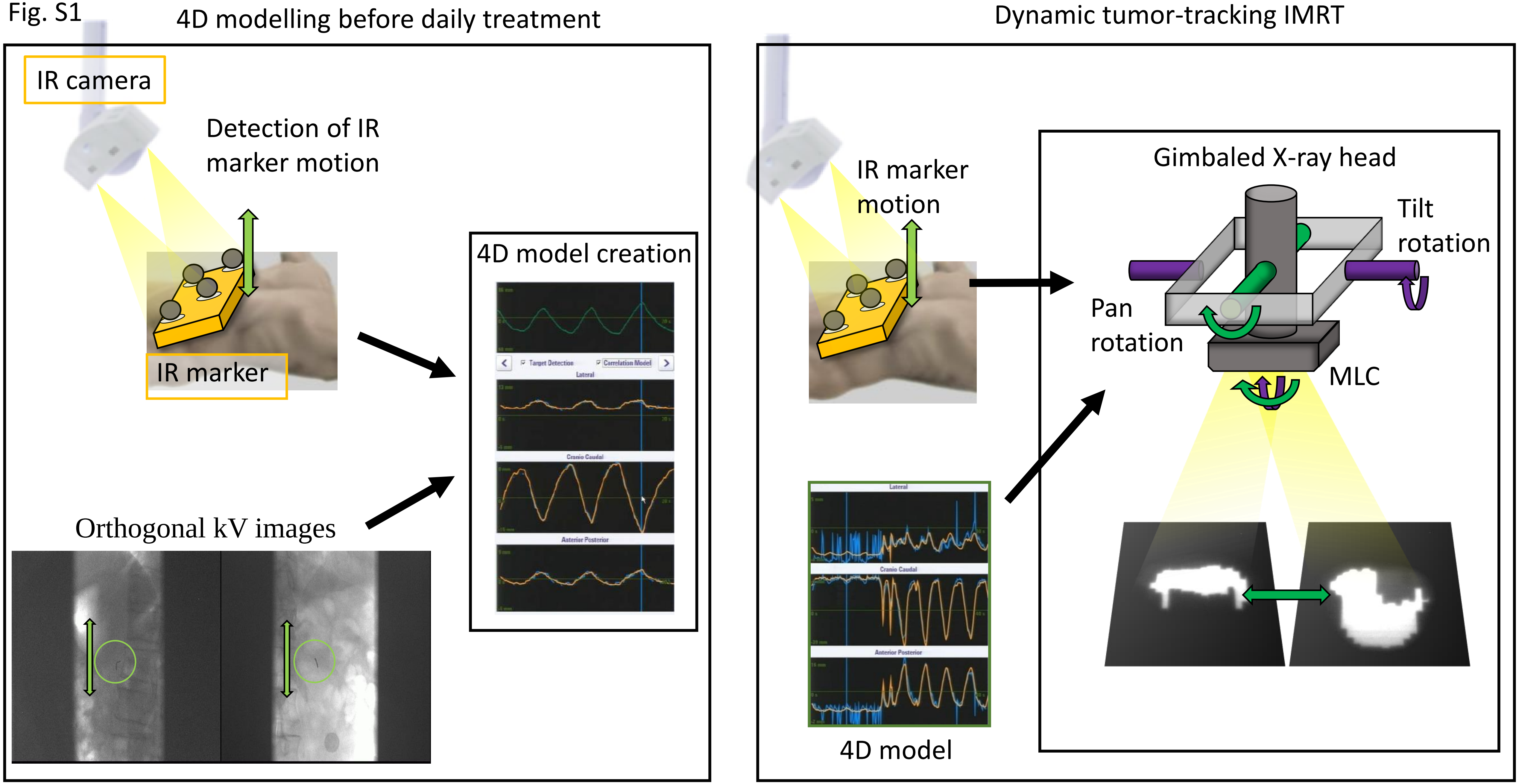
4D modelling before daily treatment
IR camera
Detection of IR
marker motion
IR marker
4D model creation
Target Detection
Correlation Model
Lateral
Cranio Caudal
Anterior Posterior
Orthogonal kV images
Dynamic tumor-tracking IMRT
IR marker
motion
Gimbaled X-ray head
Tilt
rotation
Pan
rotation
MLC
Lateral
Cranio Caudal
Anterior Posterior
4D model

Fig. S2

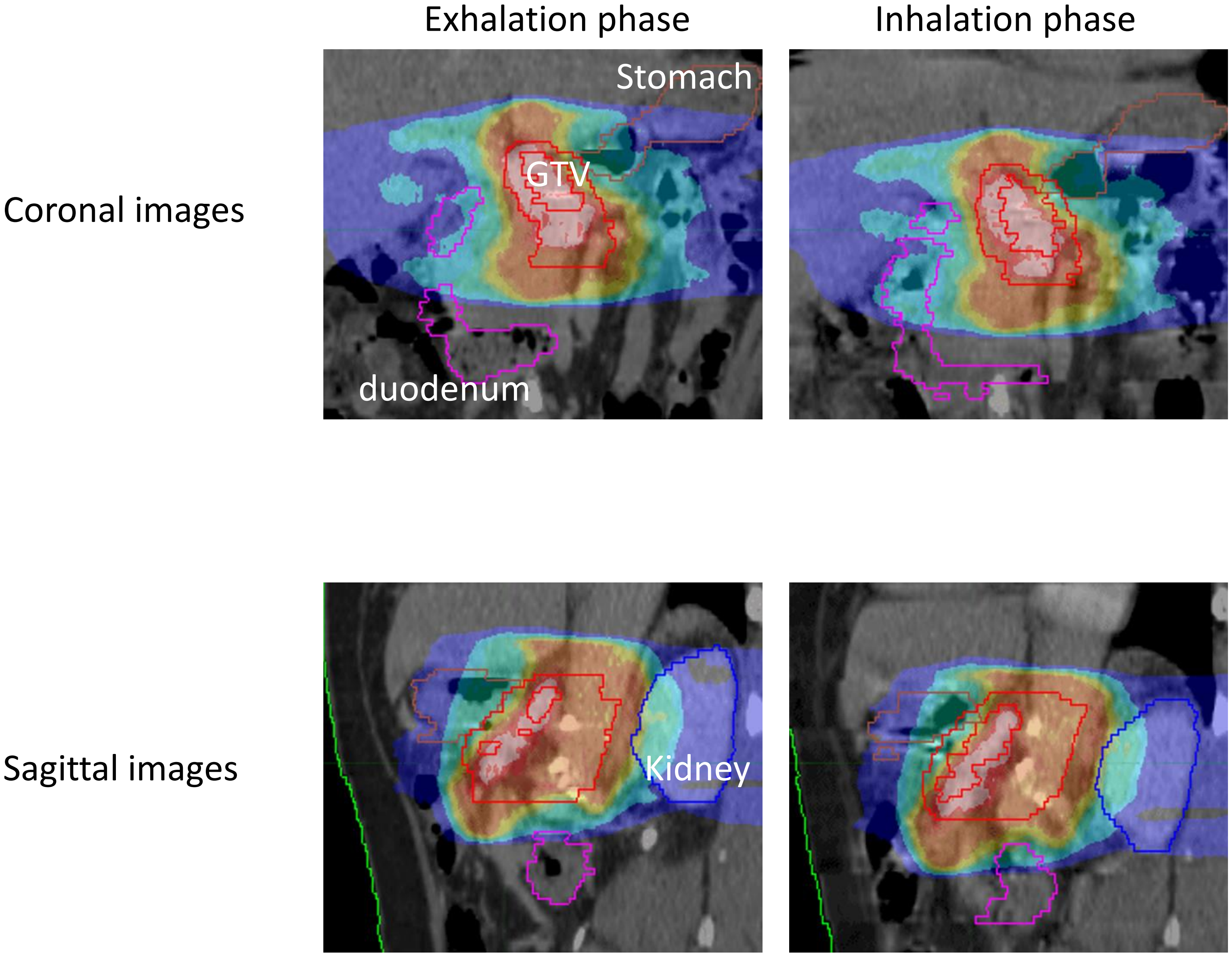
Exhalation phase
Inhalation phase
Coronal images
Sagittal images
Stomach
GTV
duodenum
Kidney

Fig. S3

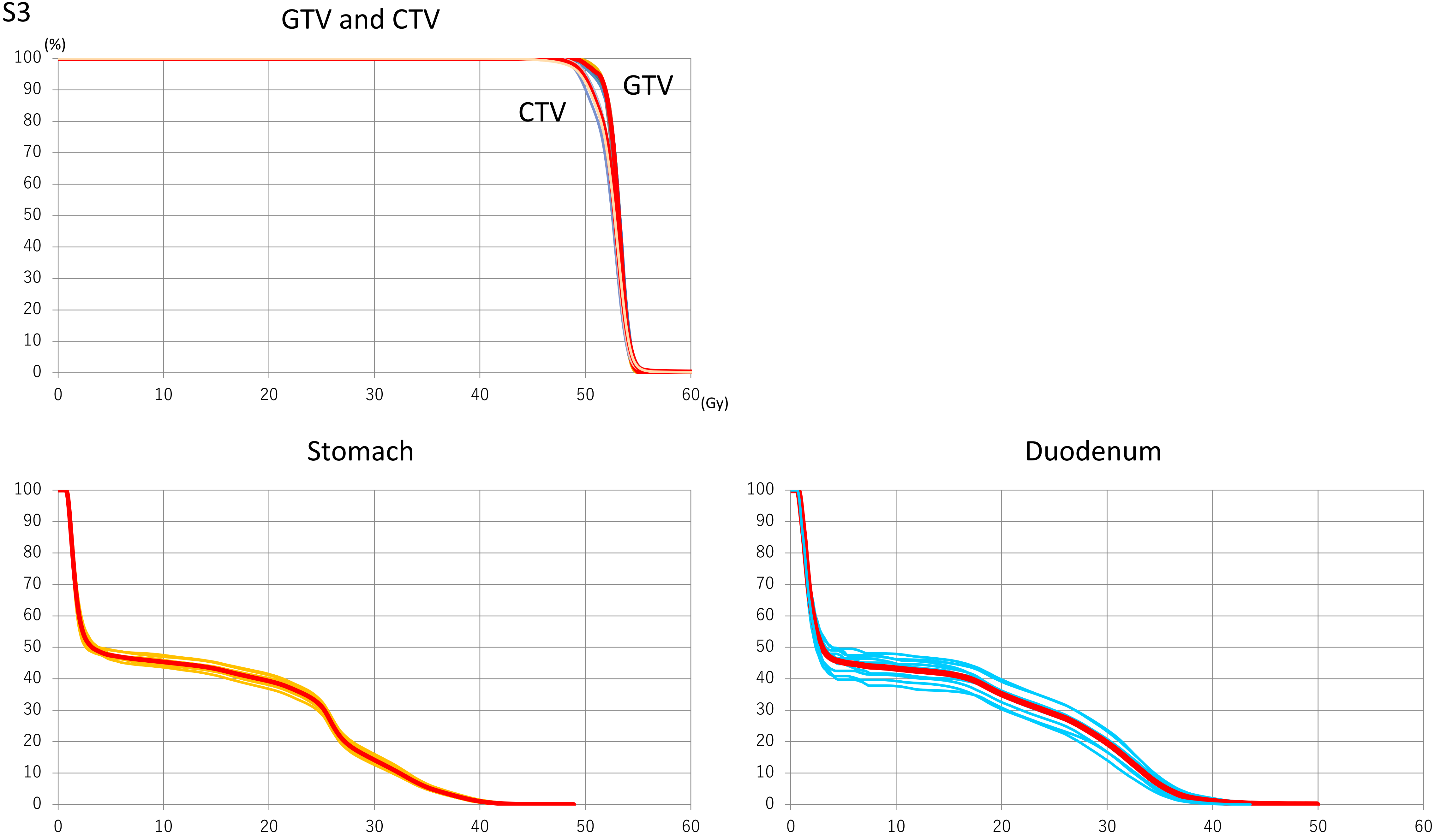

GTV and CTV
(%)
100
90
80
70
60
50
40
30
20
10
0
0
10
20
30
40
50
60
(Gy)
GTV
CTV
Stomach
Duodenum

Fig. S4

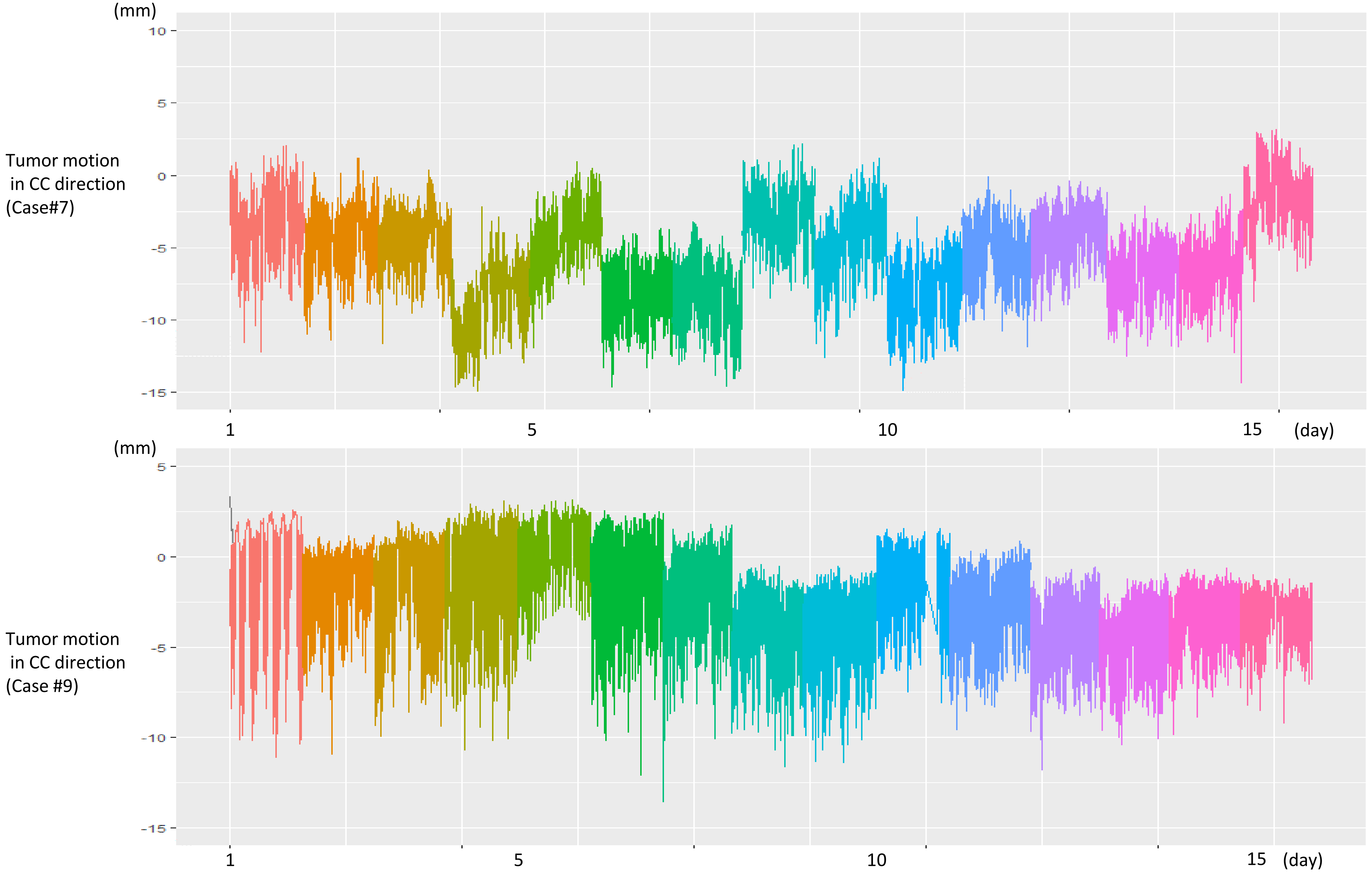
(mm)
10
5
0
-5
-10
-15
Tumor motion
in CC direction
(Case#7)
1
5
10
15
(day)
(mm)
5
0
-5
-10
-15
Tumor motion
in CC direction
(Case #9)
1
5
10
15
(day)
